# Quantitative techniques in ^18^FDG PET scanning in oncology

**DOI:** 10.1038/sj.bjc.6604330

**Published:** 2008-05-13

**Authors:** F Castell, G J R Cook

**Affiliations:** 1 Department of Radiotherapy, Royal Marsden NHS Foundation Trust, Downs Road, Sutton SM2 5PT, UK; 2 Department of Nuclear Medicine and PET, Royal Marsden NHS Foundation Trust, Downs Road, Sutton SM2 5PT, UK

**Keywords:** ^18^FDG, PET, oncology, quantitation, kinetic analysis

## Abstract

The clinical applications of ^18^F-fluoro-2-deoxyglucose (^18^FDG) positron emission tomography (PET) in oncology are becoming established. While simple static scanning techniques are used for the majority of routine clinical examinations, increasing use of PET in clinical trials to monitor treatment response with ^18^FDG and novel tracers reflecting different pharmacodynamic end points, often necessitates a more complex and quantitative analysis of radiopharmaceutical kinetics. A wide range of PET analysis techniques exist, ranging from simple visual analysis and semiquantitative methods to full dynamic studies with kinetic analysis. These methods are discussed, focusing particularly on the available methodologies that can be utilised in clinical trials.

^18^F-fluoro-2-deoxyglucose (^18^FDG) positron emission tomography (PET) is an important tool in oncology, at the forefront of functional and molecular imaging techniques. Its use has progressed from staging purposes to the assessment of response to treatment (Figure 1). The information derived from ^18^FDG PET scans is more frequently becoming the basis for decisions on subsequent management, such as the cessation or switching of chemotherapy regimes and the addition or modification of radiotherapy. It is also used clinically to establish the diagnosis of relapse following treatment.

There is a wealth of literature reporting on the role of ^18^FDG PET in malignancy, showing a wide range of associated sensitivities and specificities; frequently showing greater accuracy than other forms of conventional imaging in a number of tumour types. One of the explanations for the range of reported results may be due to the variability of scanning protocols and analytic techniques used. The analytic techniques range from simple visual qualitative assessment and semiquantitative methods, for example, standardised uptake value (SUV) measurement, to full kinetic analysis with compartmental modelling, for example, nonlinear regression analysis (NLR). A variety of analytical methods have been proposed that need to be validated for different tumour types and for assessing therapeutic response. While simple qualitative techniques are sufficient for many routine clinical examinations, an increase in the use of PET with ^18^FDG, and also novel tracers reflecting different pharmacodynamic end points in clinical trials, often necessitates a more complex and quantitative approach.

## PHYSIOLOGY OF ^18^FDG UPTAKE

Accelerated glucose metabolism is the basis for the use of ^18^FDG PET in detecting cancer. ^18^F-fluoro-2-deoxyglucose is transported into glucose-consuming cells with the aid of transporters such as GLUT-1, where it is phosphorylated by hexokinases. Overexpression of glucose transporters and hexokinases has been reported in many cancer cells, leading to their increased accumulation of phosphorylated ^18^FDG (Smith, 2001). Unless dephosphorylation occurs by glucose-6-phosphatase, ^18^FDG-6-PO_4_ remains essentially trapped in the cell. After initial accumulation, there is often rapid dephosphorylation and clearing of FDG-6-PO_4_ in tissues and organs with high glucose-6-phosphatase activity (Caraco *et al*, 2000). Malignant cells mostly show low levels of glucose-6-phosphatase compared with many normal tissues and benign pathological processes such as inflammation, leading to different kinetics of ^18^FDG accumulation between benign and malignant processes (Yamada *et al*, 1995; Lodge *et al*, 1999; Nakamoto *et al*, 2000).

## FACTORS AFFECTING ^18^F-FDG UPTAKE AND SCAN INTERPRETATION

Pathological processes other than malignancy can be responsible for some degree of initial increase in uptake, predominantly infective, inflammatory and granulomatous disorders (e.g., tuberculosis, aspergillosis, sarcoidosis, amyloidosis and postradiotherapy reactions) (Cook *et al*, 1996). This is thought to be due to uptake in the increased number of activated white cells involved in these processes and can produce a false-positive scan (Kaim *et al*, 2002). Uptake also depends on various other factors, such as body habitus, insulin levels and blood glucose (Huang, 2000). ^18^F-fluoro-2-deoxyglucose accumulation is influenced by tumour blood flow but other factors such as glucose transport and hexokinase activity are frequently rate-limiting steps, although a single factor probably does not account for ^18^FDG uptake in all tumours (Smith, 2001).

The performance characteristics of the PET scanner, scan timing and duration, measurement of injected activity and a number of other technical factors can all affect precision and accuracy of quantitative measurements. The spatial resolution of a PET scanner is usually between 5 and 8 mm in the reconstructed image. Uptake in structures less than two to three times this size can be underestimated as a result of the partial volume effect. For example, a sphere with a diameter equivalent to 1.5 times the spatial resolution will have a maximum measured activity concentration of about 60% of the true activity concentration and a mean of about 30% (Geworski *et al*, 2000). Heterogeneity of uptake in tumours (e.g., when areas of viable and necrotic tumour exist) can cause similar problems in accurate quantitation of viable tumour activity. Higher activity in surrounding tissues to the region of interest (ROI) can contribute to activity within the ROI by the spillover effect and this may also affect noninvasive measurements of arterial activity concentration (e.g., aorta or left ventricle) when used as a noninvasive method of obtaining an arterial input function (IF) for kinetic analytical methods (Cook *et al*, 1999).

The recent introduction of hybrid PET/CT (computed tomography) scanners allows combined modality acquisitions in a single sitting. The anatomical detail provided by CT allows better localisation of sites of physiologic activity and pathology identified by PET, thereby improving image interpretation and also providing rapid, low-noise transmission CT data for attenuation correction of PET emission data, essential for quantitative measurements.

A range of software platforms and packages are available for PET data processing and analysis offering different levels of flexibility and complexity to the user (Ratib, 2004). The increasing use of PET/CT has also led to the demand for better software platforms for the reconstruction and optimal display and analysis of both sets of images in a user-friendly format.

## TIME COURSE OF ^18^FDG UPTAKE AND THE OPTIMUM TIME OF IMAGE ACQUISITION

Studies have investigated uptake over time in malignant cells, some tumour-specific, for example, lung cancer and sarcomas, comparing uptake with that in benign and inflammatory lesions (Lodge *et al*, 1999; Matthies *et al*, 2002). The maximum uptake in benign tissues occurs earlier at approximately 30 min, with peak uptake in inflammatory tissues occurring at about 60 min (Yamada *et al*, 1995). There is a wider spectrum for malignant tissues, with peak uptake occurring later, sometimes up to 4 h (Lodge *et al*, 1999). More consistent and reliable measurements are likely to be possible when tumour tracer concentration has reached a plateau, as small differences in the timing of scans after injection are less likely to affect measured parameters of uptake. This creates difficulties in establishing an optimum scanning time as the half-life of the radioisotope must also be considered (110 min for ^18^F). The EORTC and NCI guidelines both recommend an optimum scanning time of 50–70 min (Young *et al*, 1999; Shankar *et al*, 2006), and this is usually the time taken for clinical scans as a best compromise between tumour to background contrast and decay of tracer.

## METHODS OF IMAGE ACQUISITION

Static emission scans are made during a period when the activity distribution is assumed to be fairly stable with a counting time long enough to obtain a good quality image, usually between 3 and 5 min on modern scanners with three-dimensional acquisition. Several bed positions (each covering between 10 and 20 cm) are required to image the whole body. Dynamic scans are carried out by acquiring a continuous series of image frames that can be of various lengths. Early frames are usually shorter to allow kinetic measurements when tracer concentrations are changing rapidly, with longer frames towards the end of a study. Dynamic emission scans are usually acquired from 0 to 60 min for ^18^FDG. One bed position only is used, limiting the area visualised. Region of interests can be placed and a tissue time–activity curve calculated. Some form of vascular IF is required for analysis for some of the more complex kinetic analytical methods. This can be derived from direct arterial sampling or indirectly by measuring activity over an arterial ROI.

## IMAGE ANALYSIS

### Qualitative analysis

Qualitative interpretation involves simple visual analysis of a static scan by the reporter. Interpretation is based on contrast in uptake between a lesion and surrounding tissues and is dependent on uptake time, blood glucose and insulin levels, and also the nature of the surrounding tissues. In small tumours with low uptake close to normal tissue with high uptake, lesions may become inconspicuous. Following therapy, normal tissue may also undergo physiological changes. For these reasons, visual subjective analysis has poor reproducibility and although it may be suitable for some routine clinical work, it is often insufficient for clinical trials where objective parameters of response are usually required and recommended (Weber, 2005; Shankar *et al*, 2006).

### Semiquantitative analysis

Tumour-to-background normal tissue ratio (T/N) requires a static image and is potentially subject to some of the weaknesses of visual analysis. Even when there has been no change in uptake in malignant tissues, a change in the normal tissue uptake post therapy can lead to a change in the T/N ratio. It is generally regarded as a less reliable index than the SUV. The SUV is the ratio between the tumour concentration of ^18^FDG and its concentration in the entire body if the tracer is uniformly distributed throughout. It has also been referred to as the differential uptake ratio or the differential absorption ratio. Its measurement requires a static, attenuation-corrected scan, accurate calibration and body weight for normalisation. Normalisation to body surface area or lean body mass may be advantageous. There is also debate about whether it should be corrected for blood glucose, as an improvement in precision has been suggested, but there is no consensus (Thie, 2004). Standardised uptake values are dependent on the uptake time but are most commonly measured at 60 min after injection. Cutoff values for differentiating between benign and malignant tissue vary among different malignancies, often lying between 2 and 3, but this may be variable for different types of tumour. Best documented is the cutoff of 2.5 used in pulmonary lesions (Lowe *et al*, 1998). There is frequently an area of overlap that can limit its use in an individual patient, some tumours not being ^18^FDG-avid and some benign processes showing high ^18^FDG uptake. It is a more reliable index to monitor change within the same patient and is reasonably precise, with less than 10% variation reported in pretreatment scans providing attention is paid in performing consecutive scans in exactly the same way (Minn *et al*, 1995).

Standardised uptake values are computationally simple and make some of the same assumptions as qualitative analysis. The measured ^18^FDG concentration is the sum of phosphorylated intracellular ^18^FDG, nonphosphorylated intracellular ^18^FDG and nonphosphorylated extracellular (intravascular and interstitial) ^18^FDG. Of these components, the first is most directly related to the metabolic activity of tumour cells. Static scans cannot differentiate between the different components and therefore do not necessarily correlate with glucose metabolic rates. They do not take into account the physiological changes affecting the availability of ^18^FDG. This may be of particular importance in assessing response to therapy, when there may be changes in the distribution of ^18^FDG throughout the body and also in the uptake by the lesion.

Dual-point scanning often uses semiquantitative indices such as SUV but exploits the different kinetics between benign and malignant tissue by comparing activity at two time points, usually at 60 and 120 min, with a number of studies reporting better tissue characterisation when benign and malignant lesions exist (Yamada *et al*, 1995; Nakamoto *et al*, 2000; Matthies *et al*, 2002; Ratib, 2004).

### Quantitative analysis

#### Simplified kinetic analysis

Simplified kinetic analysis is a generic term for methods that attempt to estimate tumour glucose metabolic rate without the need for full dynamic studies or arterial blood measurements but at the same time overcoming some of the limitations of SUVs. They usually rely on a single time point (SKA-S) static image and a single timed blood sample (Hunter *et al*, 1996), but, more recently, multiple time point (SKA-M) methods have been proposed (Sundaram *et al*, 2004) with reports of lower variability and bias compared with SKA-S.

#### Kinetic analysis

Full quantitative analysis uses kinetic modelling approaches to derive the metabolic rate for glucose (MR_glu_) or ^18^FDG (MR_FDG_). Dynamic studies follow metabolic activity over a longer period of time, using a more complex model of the underlying physiology and seeking to remove more of the effects of confounding factors. They are less dependent on the time of measurement. The kinetic models are based on the concept of several compartments that contain ^18^FDG, linked by kinetic processes that provide a mechanism of exchange of ^18^FDG (Willemsen and Van den Hoff, 2002). Rate constants (*k*) describe the rate of movement of ^18^FDG between compartments. These are assumed to represent specific physiological processes such as blood flow, glucose metabolism and enzyme activity. The transport of ^18^FDG across the capillary/cellular membrane is accounted for by the rate constants *K*_1_ and *k*_2_ (forward and reverse transport). The phosphorylation of ^18^FDG to ^18^FDG-6-PO_4_ is represented by *k*_3_ and sometimes a small dephosphorylation rate constant, *k*_4_, for the dephosphorylation of ^18^FDG. *K*_*i*_ (*K*_1_*k*_3_/(*k*_2_+*k*_3_)) is the net influx rate constant for ^18^FDG. Certain assumptions have to be made related to the biological system and ^18^FDG and to the specific experimental procedure employed to make the measurements. The processes of the biological system influencing the kinetic behaviour of ^18^FDG are assumed to be in steady state, although the ^18^FDG concentration itself does not have to be in a steady state. The data in dynamic studies are obtained from a series of image frames as described earlier. An IF is measured from either arterial or arterialised venous blood sampling. More recently, noninvasive measurement of arterial activity in the aorta or left ventricle dynamic image data has been validated (Hoekstra *et al*, 2002).

Nonlinear regression analysis uses an algorithm to derive values for the rate constants and also a blood volume term. It uses a two-tissue compartmental model with an arterial plasma IF and tissue time–activity data (tissue TAC) over 0–60 min to measure *K*_*i*_. It provides one of the most accurate estimates of tumour glucose use (Dimitrakopoulou-Strauss *et al*, 2002). In clinical practice, low count statistics and artefacts attributable to patient movement can affect its accuracy.

Patlak graphical analysis requires fewer image frames than NLR but still requires a blood IF (Patlak *et al*, 1983). A tissue TAC is provided from the dynamic image data and the net metabolic clearance of ^18^FDG can then be calculated. The rate of uptake from the plasma is given by the slope of the linear portion of the plot. *K*_*i*_ can therefore be derived and the tissue metabolic rate of glucose is calculated. This method assumes that *k*_4_=0, that is, irreversible trapping of ^18^FDG-6-PO_4_. Patlak analysis has been shown to approach the accuracy of NLR but is computationally simpler and considered less susceptible to image noise (Weber, 2005).

## CLINICAL APPLICATIONS

### Tissue differentiation and tumour grading

The initial role of ^18^FDG PET was in the differentiation between benign and malignant lesions, especially in indeterminate solitary pulmonary nodules (Lowe *et al*, 1998) with further exploration into grading and prognosis assessment of various tumours (Dimitrakopoulou-Strauss *et al*, 2001; Shimoda *et al*, 2007). Standardised uptake values have been shown to be reliable in differentiating between benign and malignant lesions in a number of situations, but in others visual analysis has been reported to be equivalent (Lowe *et al*, 1994). It is possible that visual analysis may often be sufficient with an experienced interpreter. A number of studies have shown that various forms of kinetic analysis have better specificity (Dimitrakopoulou-Strauss *et al*, 2002) with improved performance in lesions with borderline SUVs but at the expense of greater scan acquisition and analysis complexity. There is less evidence on the use of PET to determine grade of malignancy, with few dynamic studies reported. However, there is some evidence that ^18^FDG PET correlates with tumour grade and infers prognosis in some tumour types, examples having been reported in bone, breast and brain tumours, among others (Dimitrakopoulou-Strauss *et al*, 2001; Borbely *et al*, 2006; Shimoda *et al*, 2007).

### Assessment of response to treatment and treatment modification

Several studies have demonstrated the ability to predict clinical or pathological response at an early time point by measuring changes in ^18^FDG tumour activity and to infer a better prognosis in responders. The results of such studies imply that early changes in tumour metabolism predict subsequent response and correlate with outcome. The optimum time point for assessment is likely to be variable, depending on individual tumours and their speed of response to different treatments with variation in studies reflecting different research questions. Wahl *et al* (1993) showed a reduction in SUV and net ^18^FDG influx as early as 8 days after starting chemohormonotherapy for primary breast cancer, which predated changes in size and predicted ultimate pathological response. Similarly, Weber *et al* (2001) have shown that a greater than 35% reduction in SUV in gastro-oesophageal cancers after one cycle of chemotherapy predicts pathological response and increased time to progression and overall survival. Interim response assessment is also being used after one or two cycles of chemotherapy to inform subsequent management, completing therapy in responding patients but intensifying or changing treatment in nonresponders. This approach has been most extensively investigated in lymphoma (Israel *et al*, 2004).

There is general agreement that at least semiquantitative techniques are superior to qualitative assessment alone in monitoring treatment response (Young *et al*, 1999; Hoekstra *et al*, 2000; Weber, 2005; Shankar *et al*, 2006) and are probably sufficient for most routine clinical work. These methods may be limited where maximal tumour response has not been achieved or when there is an accompanying inflammatory response associated with ^18^FDG activity. The careful timing of scans after injection is also crucial to ensure maximal precision between scans.

A number of parameters may be affected by the metabolic changes that occur as a result of therapy, such as plasma clearance of ^18^FDG secondary to reduced renal function. This can affect the assumptions made in interpretations of data, including that the contribution of nonphosphorylated ^18^FDG to the total signal can be ignored. This can result in the poorer performance of qualitative and semiquantitative analysis. Weber (2005) suggested that although changes in SUV are generally well correlated with changes in net influx rate (*K*_*i*_), this may not apply in tumours with relatively low metabolic activity. In this situation, the contribution of nonphosphorylated ^18^FDG to the total ^18^FDG signal cannot be neglected, and therapy-induced changes in *K*_*i*_ may correlate less well with changes in SUV. Because of these and other described limitations of semiquantitative techniques, there will remain a role for more complex and invasive dynamic scan acquisitions and kinetic analysis to allow for better discrimination of responders from nonresponders (Hoekstra *et al*, 2002), particularly in the phase 1 trial setting or when novel tracers measuring different pharmacodynamic end points are being used where less knowledge exists on the kinetics of uptake. Compartmental modelling of dynamic data is often the preferred form of analysis for pharmacokinetic measurements of novel drugs as it allows a detailed measure of the individual kinetic processes involved (Willemsen and Van den Hoff, 2002).

## CONCLUSION

Positron emission tomography has become an important part of the management of cancer. Qualitative and semiquantitative interpretation of static scans is probably sufficient for routine clinical staging purposes. In lesions of uncertain significance, semiquantitative techniques (e.g., SUV) may help guide interpretation, although due to variability in ^18^FDG avidity between different tumour types, may be limited in discrimination between benign and malignant tissue in an individual case. For the assessment of response to therapy, further studies are required. Although semiquantitative measures usually suffice in the clinic, it may be that the use of dynamic scanning with kinetic analysis is more appropriate for drug development trials and may provide a more accurate assessment of changes in tumour metabolism. This may be worth the additional time and effort to perform as patient treatment becomes more tailored to the individual, based on their response to treatment received so far. However, although kinetic approaches offer improved accuracy and discrimination, they are practically more complex and validation of semiquantitative static or simplified dynamic techniques would be particularly attractive in this role.

## Figures and Tables

**Figure 1:**
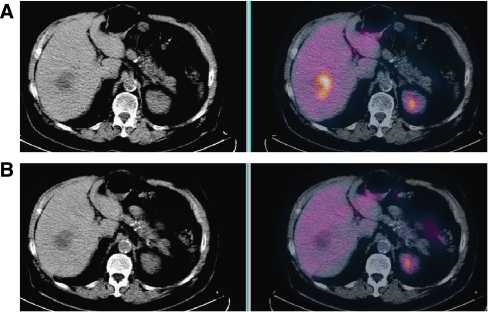
A liver metastasis from a gastric GIST before (**A**) and 1 week after (**B**) commencing imatinib therapy. FDG PET/CT scans, unenhanced CT (left), fused FDG PET and CT (right). Although there has been no morphological change in the metastasis, the abnormal baseline metabolic activity (colour scale) has rapidly resolved indicating sensitivity to the drug. The SUV fell from 5.0 to 1.8.
